# Use of real-time cellular analysis and Plackett-Burman design to develop the serum-free media for PC-3 prostate cancer cells

**DOI:** 10.1371/journal.pone.0185470

**Published:** 2017-09-25

**Authors:** Ai Zhao, Fahai Chen, Chunhong Ning, Haiming Wu, Huanfang Song, Yanqing Wu, Rong Chen, Kaihua Zhou, Xiaoling Xu, Yinxiang Lu, Jimin Gao

**Affiliations:** 1 Zhejiang Provincial Key Laboratory for Technology & Application of Model Organisms, School of Laboratory Medicine& Life Sciences, Wenzhou Medical University, Wenzhou, Zhejiang, China; 2 Hospital 212 of the Nuclear Industry, Wuwei, Gansu, China; Universiti Putra Malaysia, MALAYSIA

## Abstract

In this study, we developed a rapid strategy to screen a serum-free medium for culturing the anchorage-dependent PC-3 prostate cancer cells, which was going to be prepared in large scale to generate GM-CSF/TNFα-surface-modified whole cell prostate cancer vaccine. Automated real-time cellular analysis as a rapid and non-invasive technology was used to monitor the growth of PC-3 cells in 16-well plates. At the same time, Plackett-Burman design was employed to identify the most influential formulation by integrating relevant information statistically. The effects of the 16 selected factors were evaluated during exponential cell growth and three medium constituents (EGF, FGF and linoleic acid) were identified to have significant effects on the cell growth. Subsequently, the response surface methodology with central composite design was applied to determine the interactions among the three factors so that these factors were optimized to improve cell growth. Finally, the prediction of the best combination was made under the maximal response to optimize cell growth by Design-Expert software 7.0. A total of 20 experiments were conducted to construct a quadratic model and a second-order polynomial equation. With the optimized combination validated by the stability test of serial passaging PC-3 cells, the serum-free medium had similar cell density and cell viability to the original serum medium. In summary, this high-throughput scheme minimized the screening time and may thus provide a new platform to efficiently develop the serum-free media for adherent cells.

## Introduction

Mammalian cell cultures are often used to make biological products such as monoclonal antibodies, recombinant proteins and vaccines. At the same time, mammalian cells are widely used to treat the patients with various types of cancers and viral infections, and their effectiveness have been well demonstrated [1]. Therapeutic cancer vaccines are usually aimed at tumor-specific or tumor-associated antigens to induce active immune response against tumor [2]. The first therapeutic cancer vaccine of prostate cancer approved by FDA is *Sipuleucel-T*, which is essentially an autologous ex-vivo dendritic cell-based vaccine, pulsed with a single antigen of prostatic acid phosphatase (PAP) fused to GM-CSF [3, 4]. Our novel therapeutic prostate cancer vaccine has been developed by use of GM-CSF/TNFα surface-modified PC-3 cells to treat the patients with advanced prostate cancer [5], and thus large-scale production of anchorage-dependent PC-3 prostate cancer cells will be required for vaccine manufacturing.

Mammalian cells are typically cultured in growth medium supplemented with 1% to 20% fetal bovine serum rich in the nutrients necessary for cell growth such as growth factors, amino acids and hormones. However, the use of serum raises a series of potential risks of contamination by animal-derived materials such as viruses. To address the issue, many kinds of materials have been used to replace the role of serum in cell culture. Early serum-free media are complex, because they contain undefined raw materials derived from plant or other non-animal sources such as hydrolysates (peptones of soy, wheat, yeast, etc.) and proteins (bovine serum albumin, transferrin, etc.) [6–9]. Therefore, current medium development has been focusing on the chemically defined media.

The medium optimization is an important step in the process development of cell culture, it determines cell growth or productivity. In addition, the medium components have some significant effects on the expression of secreted proteins. The traditional strategy used for the medium optimization is laborious and time-consuming [10–14]. Therefore, new technologies for medium development are increasingly dependent on the accelerated screening and optimization of many conditions. Automated real-time cellular analysis (RTCA) in combination with factorial experimental design provides the opportunity to explore a large design space and is thus cost-effective by saving raw materials, culture media, labor and time. In this study, we described a novel experiment design for the medium development of a fed-batch cell culture of PC-3 prostate cancer cells. As a rapid, real-time and non-invasive technique, RTCA was applied to monitor the culture of adherent cells in 16-well plates [11, 12]. Plackett-Burman design (PBD) was employed to screen influential factors from many kinds of constituents of the cell culture supplements [13, 14]. Subsequently, response surface methodology (RSM) in combination with central composite design (CCD) was used to optimize the most influential factors and subsequently investigate the interactions among the factors [15, 16].

## Materials and methods

### Cell line and culture conditions

The prostate cancer cell line PC-3 used was purchased from Shanghai cell bank of Chinese academy of sciences. The PC-3 cells were cultured at DMDM/F12 with 5% FBS and 37°C, 5% CO_2_ according to the ATCC instruction. Experiments for screening assays were performed in 16-well E-plates seeded with cell densities ranging from 2 to 4*10^5^ cells ml^-1^ in a volume of 200μl per well. Cells were all seeded at the exponential growth phase of the amplification culture. PC-3 cells were washed by PBS and followed by 1000 RPM centrifugation for 5 min before screening assay, and then suspended in PBS as stock solution. Each tested condition was performed in duplicate and each experiment was done twice.

The following 16 independent variables were selected for analysis [1, 8, 9, 17–29]: insulin (5mg/l), transferrin, sodium selenite, sodium L-ascorbate, ferric citrate, L-glutathione, bovine serum albumin (BSA), EGF, FGF, ethanolamine, linoleic acid, arachidonate, thioglycerol, hydrocortisone, yeast hydrolysate, penicillin-streptomycin solution and succinic acid (*Sigma*, Shanghai). The 16 additives in the serum-free medium were selected through the biological function of each additive and the characteristics of the cells [1, 8, 9, 17–29]. For example, Protein or yeast hydrolysate is rich in peptides, nucleic acids, vitamins and trace elements, but not essential ingredients in the serum-free culture [1, 8, 9]. Insulin, sodium selenite and transferrin are required for almost all kinds of cells to grow in serum-free medium [17–21]. Insulin promotes the synthesis of RNA, protein and fatty acids, and inhibits apoptosis [19]. There are specific transferrin receptors on most mammalian cells, and the transferrin Fe3^+^ complex is the main source of essential iron for the cells. In addition, transferrin has growth-factor-like nature and can be used in combination with other trace elements such as vanadium. However, the role of transferrin in serum-free medium may be replaced by a ferric salt, such as iron citrate, ferrocuanic acid or iron gluconate. Selenium is necessary for the activity of glutathione peroxidase [22]. Albumin is a commonly added factor in serum-free medium by stabilizing and regulating the activities of vitamins, lipids, hormones, metal ions and growth factors, as well as by binding to toxins and reducing the effect of proteases on cells in serum-free culture [23]. Some cells in serum-free culture also need some low molecular weight nutrients such as trace elements, vitamins, lipids, etc [24]. Vitamins C and E are antioxidants. Succinic acid provides the required lipid for cell membrane synthesis and the water-soluble lipids for cell growth [25, 26]. Growth factors are supplemental factors which are necessary to maintain the survival, proliferation and differentiation of cells in vitro. The most common growth factors in serum-free medium are epidermal growth factor (EGF) and fibroblast growth factor (FGF) [17, 27]. PC-3 cells over-express EGF receptor (EGFR), which is involved in promoting tumor hyperplasia and androgen stimulation [28, 29].

The concentrations of chosen variables are given in Table 1. The concentration of each additive was determined by referring to many previous reports [1, 9, 30–32]. For example, the KDMEM was supplemented with 5% (v/v) KSR, 12 ng/ml bFGF, 5 ng/ml EGFand 1 lg/ml hydrocortisone supported sufficient proliferation of aHDFs for 1 week [1]. Rrice straw(5.0, 30.0 g/L), K2HPO4 (0.5 5.0 g/L), KH2PO4 (0.1, 1.0g/L), MgSO4.7H2O (0.1, 1.0g/L), CaCl2.2H2O (0.1, 1.0 g/L), Tween 80 (0.05, 1.0g/L), NaCl (0.1, 1.0g/L), Yeast extract (1.0, 10.0 g/L), (NH4)2SO4 (0.5, 5.0g/L) to was added to optimize cellulose-free xylanase production Thermophilic Streptomyces thermovulgaris TISTR1984 [30]. RPMI1640 was supplemented with bovine serum albumin 2.5 g/l, insulin 5 mg/l, ferric citrate 2 mg/l, ethanolamine 1.22 mg/l, linoleic acid 1 mg/l, oleic acid 1 mg/l, palmitic acid 1 mg/l, L-glutamine 584 mg/l, sodium pyruvate 110 mg, 2-mercaptoethanol 0.78 mg/l, 1-thioglycerol 5.41 mg/l, nonessential amino acids 20 ml/l, vitamins solution 10 ml/l to develop a serum-free medium for in vitro expansion of human cytotoxic T lymphocytes [31]. The growth factors such as EGF and FGF added in the serum-free medium and their concentrations were adopted from the references [9, 15, 16, 32].

**Table 1:** Factors and their corresponding concentrations by Plakett-Burman design.

Factors	Variables	Concentration	
		Low(-1)	High(1)
**A**	Transferrin	3.8(mg/L)	7.5(mg/L)
**B**	Sodium selenite	0.005(mg/L)	0.01(mg/L)
**C**	Sodium L-ascorbate	37.5(mg/L)	75(mg/L)
**D**	Ferric citrate	2.5(mg/L)	5(mg/L)
**E**	L-glutathione	0.5(mg/L)	1(mg/L)
**F**	BSA	1(g/L)	2(g/L)
**G**	EGF	10(ng/ml)	20(ng/ml)
**H**	FGF	10(ng/ml)	20(ng/ml)
**I**	Ethanolamine	2(mg/L)	4(mg/L)
**J**	Linoleic acid	1(mg/L)	2(mg/L)
**K**	Arachidonate	1(mg/L)	2(mg/L)
**L**	Thioglycerol	3(mg/L)	6(mg/L)
**M**	Hydrocortisone	1(ng/ml)	2(ng/ml)
**N**	Yeast hydrolyzate	1(ml/L)	2(ml/L)
**O**	Penicillin-Streptomycin Solution	50(μg/ml)	100(μg/ml)
**P**	Succinic Acid	0.5(μg/ml)	1(μg/ml)

### Cell counting

The PC-3 cells was dissociated by trypsinization and suspended in PBS. The cells suspension was dyed by 0.4% trypan blue, and then 20μl was used for cell counting. The viable cell density was measured through automatic cell counter (*Countstar BioMed*, Shanghai) and trypan blue dye exclusion with 10% accuracy.

### RTCA detection of cell proliferation

The impedence-based biosensor system was used to measure cell viability, growth and proliferation by *xCELLigence* RTCA (*Roche*, Shanghai). The changes of cell morphology and behavior were continuously monitored in real time, non-invasive and dynamic through microelectronics located in the wells of RTCA E-plates. The impedance signal was converted in the arbitrary cell index (CI) unit and recorded on the external computer, and the RTCA software was used to analyze the data [11,12].

Accordingly, the media were added into 16-well E-plates with 150μl per well and monitored to ensure the right base-line. Then, the stock solution of PC-3 cells was seeded with 4*10^5^ cells ml^-1^(in 150ul), and kept on the E-plate for 30min at room temperature. The monitoring continued (every 1h) for another 72h under normal incubation conditions (37°C, 5% CO_2_ atmosphere). Each sampling was done in duplicate.

### Screening for essential medium components by use of PBD

PBD was employed to screen the most significant medium components for cell growth. Design-Expert software 7.0 (*Stat-Ease*, Minneapolis) was used for the experimental designs and subsequent data analysis. PBD could be applied to investigate up to N-1variables with N experiments, which was a two-level fractional design with high(+1) and low(-1) levels and widely used to screen the most significant factors from lots of varies [13, 33]. All the experiments were carried out according to the design matrix, which was based on the DMEM/F12 medium. The matrix was shown in Table 2. Each row represented different nutrient concentrations, each independent variable was tested at two levels, a high(+) and a low(-) level. The slope values (Y) were obtained as responses from RTCA by the method of the least squares to fit the first-order mode.

**Table 2:** Plackett-Burman matrix of the experimental design.

	A	B	C	D	E	F	G	H	I	J	K	L	M	N	O	P	Q	R	S	T	Y
**1**	-1	1	-1	-1	-1	-1	1	1	-1	1	1	-1	-1	1	1	1	1	-1	1	-1	35
**2**	1	-1	-1	-1	-1	1	1	-1	1	1	-1	-1	1	1	1	1	-1	1	-1	1	28.4
**3**	-1	-1	-1	1	1	-1	-1	1	-1	-1	1	1	1	1	-1	1	-1	1	-1	-1	29.7
**4**	-1	-1	-1	-1	-1	1	-1	-1	-1	-1	-1	-1	-1	-1	-1	-1	-1	-1	-1	-1	33.8
**5**	-1	-1	1	1	1	-1	-1	1	-1	1	-1	-1	-1	-1	1	1	-1	1	1	-1	32.1
**6**	1	1	1	1	-1	1	-1	1	-1	-1	-1	-1	1	1	-1	1	1	-1	-1	1	33
**7**	-1	-1	-1	-1	1	-1	1	1	1	-1	-1	1	1	1	1	-1	1	-1	1	-1	28.9
**8**	1	1	-1	-1	-1	1	-1	1	-1	1	-1	1	-1	-1	-1	-1	1	1	-1	1	33.4
**9**	1	-1	1	-1	1	-1	1	-1	-1	1	1	-1	1	1	-1	-1	1	1	1	1	36.1
**10**	1	1	1	-1	1	1	-1	-1	-1	-1	-1	1	1	-1	1	1	-1	-1	-1	1	31.9
**11**	1	1	-1	1	1	-1	1	1	1	1	1	-1	1	-1	1	-1	-1	-1	1	1	32.3
**12**	-1	1	1	1	1	1	-1	-1	1	-1	-1	-1	1	1	1	-1	1	1	1	-1	32.5
**13**	-1	-1	1	1	1	-1	1	-1	-1	1	1	1	1	1	1	-1	1	-1	-1	-1	41.4
**14**	1	-1	-1	1	1	1	-1	-1	1	-1	1	-1	-1	-1	-1	1	1	-1	-1	1	34.6
**15**	-1	1	1	-1	-1	-1	1	-1	1	1	1	1	-1	-1	-1	1	-1	-1	-1	-1	32.7
**16**	1	1	-1	1	1	1	1	-1	-1	-1	1	1	-1	1	1	-1	-1	1	1	1	36.1
**17**	-1	1	1	-1	1	-1	1	1	1	1	1	-1	1	-1	-1	-1	-1	1	-1	-1	31.4
**18**	-1	1	1	-1	-1	-1	-1	-1	1	-1	-1	1	1	-1	-1	1	1	1	-1	-1	31.3
**19**	1	-1	1	1	-1	-1	-1	-1	1	1	1	1	-1	1	-1	-1	-1	-1	1	1	32
**20**	1	-1	1	-1	-1	-1	-1	1	1	-1	1	1	-1	-1	1	1	1	1	-1	1	28

Letters from A to T are dummy variable. +, high level of a particular variable; -, low level of the same variable.

### Optimization of screened factors by RSM

RSM in combination with CCD was used to optimize the selected medium constituents. RSM combined mathematical and numerical techniques so as to make it easy for modeling and analysis. RSM, which was useful for numerous variables influencing the response and the objective, was applied to optimize the response [15, 16, 34]. CCD was the most widely-used design for RSM. According to the corresponding experimental table, the data were analyzed through quadratic regression fitting with the quadratic equation, and the factors of the main effects and interaction effects were studied according to the response value maximization under the condition of optimal concentration of each factor. After regression fitting, the factors as a function of response values could be expressed in the following quadratic equation:

$$Y=\beta_{0}+\beta_{1}X_{1}+\beta_{2}X_{2}+\beta_{3}X_{3}+\beta_{12}X_{1}X_{2}+\beta_{13}X_{1}X_{3}+\beta_{23}X_{2}X_{3}+\beta_{11}X_{12}+\beta_{22}X_{22}+\beta_{33}X_{32}$$

Y represents the response value type, beta represents the regression coefficient, x1, x2, x3 represents the main influencing factors. The nature of the polynomial fitting model equation is expressed by determination coefficient R2, and the statistical significance is determined by F test. The three medium factors (independent variables) were studied at five different levels (Table 3). CCD was designed in the 2^k^ factorial design or department analysis for design on the basis of up to 2^k^ axis point (plus or minus a, 0,…, 0), (0, +a,…,0), (0, 0,…, plus or minus a) center and N(0, 0,…) composition. For three factors (k = 3), each factor took five levels. A value made CCD rotating, and thus the experimental design under the condition of the optimal value was given in priority to provide the precision of estimation, such as in all directions and by adjusting the center of Times. N could make CCD with orthogonality and generality. CCD design was good for 2 to 5 factors with 5-level optimization [13, 14, 35, 36].

**Table 3:** Experimental design and results by central composite design.

	X1	X2	X3	Y
**1**	1.682	0.000	0.000	44.4
**2**	0.000	0.000	0.000	48.7
**3**	0.000	0.000	0.000	46.9
**4**	-1.000	-1.000	1.000	31.7
**5**	0.000	-1.682	0.000	21.5
**6**	-1.682	1.000	1.000	35.6
**7**	-1.000	0.000	0.000	55.4
**8**	1.000	1.000	1.000	36.6
**9**	0.000	0.000	0.000	46.6
**10**	0.000	0.000	1.682	24.7
**11**	0.000	0.000	0.000	48
**12**	-1.000	-1.000	-1.000	35.6
**13**	1.000	-1.000	-1.000	25.9
**14**	1.000	-1.000	1.000	26.2
**15**	0.000	0.000	0.000	45.6
**16**	0.000	0.000	-1.682	26.4
**17**	0.000	0.000	0.000	47.5
**18**	0.000	1.682	0.000	31.7
**19**	1.000	1.000	-1.000	33.8
**20**	-1.000	1.000	-1.000	35.6

X1, X2 and X3 represent concentration levels of EGF, FGF and linoleic acid, respectively. Y represents the response value.

### Determination of specific growth rate

The computational formula:

$$\mu =\frac{\ln X_{n}-\ln X_{n-1}}{t_{n}-t_{n-1}}$$

μ: cell-specific growth rate;
X_n_,X_n—1_: two consecutive sampling of density of living cells (cell/mL);
t_n_ and t_n—1_: two consecutive sampling time-points (d).

### Measurement of glucose and lactic acid concentrations

PC-3 cells with indicated inoculation density of 2–3*10^5^ cell/ml and indicated volume of cultivation were seeded in six orifice under normal culture situation. The supernatant was used for the determination of glucose and lactic acid concentrations by use of the kits (*Sangon Biotech*, Shanghai) after 24h and 48h.

### Statistical methodology

Design-Expert software 7.0 was used to analyze each output of response values, generating reduced quadratic mixture models for each one and at each time point. The analysis of variance (ANOVA) of each model indicated the main factors with a significant influence. Models were the used to predict best mixtures from the 20 formulations to maximize both growth and production.

## Results

### Analysis of the characteristics of the cell metabolism and proliferation

**Fig 1:**
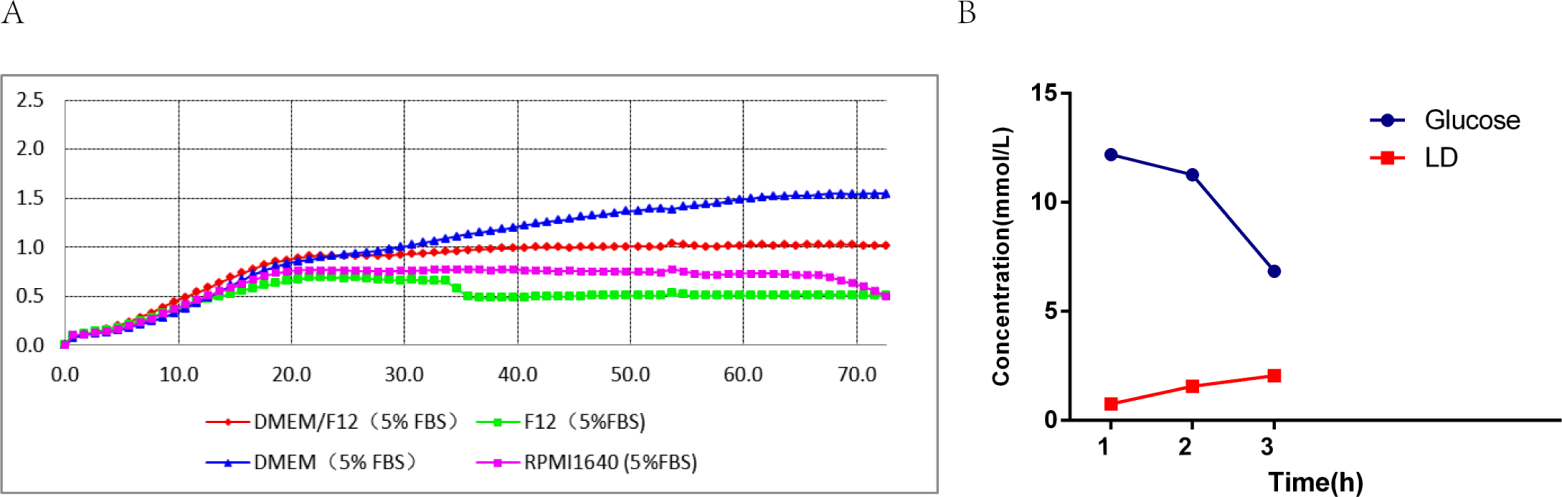
Basal medium screening. Four basic media were screened: DMDM (5%FBS), DMEM/F12 (5%FBS), F12 (5%FBS), RPMI1640 (5%FBS).

The proliferation rate of PC-3 cells was studied in four different media (DMEM, DMEM/F12, RPMI1640, F12 with 5% FBS) from 0h to 72h. The cell indices represented cell density. The proliferation rates of PC-3 cells in the four media exhibited the upward trends during the culturing period. As shown in Fig 1A, there were similar proliferation trends in all curves in the first 20h, but the trend of proliferation rates appeared different after 20h. The curve of DMEM maintained a rapid growth, and the curves of other three media reached the plateaus. However, the cell index of MEM/F12 was higher than that of RPMI1640 or F12, indicating that PC-3 cells grew better in DMEM or DMEM/F12 with 5% FBS than in RPMI1640 or F12 with 5% FBS. In addition, as shown in Fig 1B, the glucose and lactate metabolic analysis of PC-3 cells showed that the glucose concentration in the media was decreased from 12.19 mmol/l to 6.62 mmol/l after 48 h, while the lactic acid concentration increased from 0.76 mmol/l to 2.06 mmol/l, indicating that PC-3 cells consumed much glucose when they proliferated rapidly. Therefore, we selected DMEM/F12 medium containing high glucose as the basis for the medium optimization.

**Fig 2:**
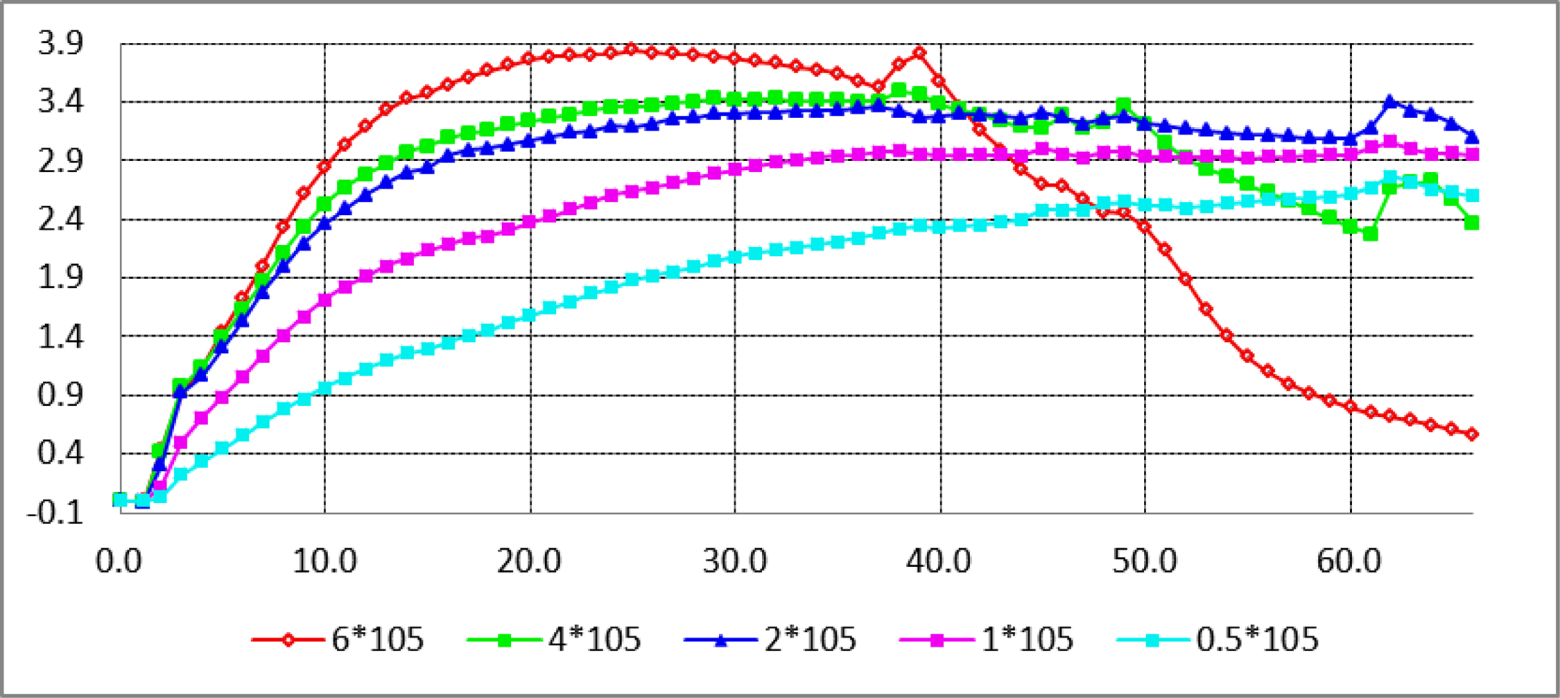
PC-3 cell proliferation curves at different seeding densities. In order to determine the optimal innoculation density of PC-3 cells, we adopted five cell seeding densities such as 6*10^5^, 4*10^5^, 2*10^5^, 1*10^5^, and 0.5*10^5^ cell/ml.

In order to determine the characteristics of PC-3 cells, we adopted five cell seeding densities such as 6*10^5^, 4*10^5^, 2*10^5^, 1*10^5^ and 0.5*10^5^ cell/ml. Every cell seeding density was tested in triplicate. As showed in Fig 2, under different seeding densities the cell indices displayed a S type of cellular proliferation characteristics during the culturing period. Under the condition of 6*10^5^ seeding density, the cell index exhibited a rapid growth, but declined quickly after a short plateau. Under the condition of 4*10^5^cell/ml or 2*10^5^ cell/ml seeding density, the cell indices displayed similar trends, indicating slower growth than that at 6*10^5^ cell/ml seeding density. Under the condition of 0.5*10^5^ cell/ml, the cell index had a slowest growth. Considering that PC-3 cells from serum culture into serum-free culture had a process of adaptation and pressure selection so that excessive seeding density could lead to a short cell proliferation cycle, we selected 2–5*10^5^ cell/ml seeding density for subsequent experiments.

### Screening essential medium components by use of PBD

PBD was widely used to screen the most significantly independent variables with a few runs. Here, the total of 16 factors were selected as independent variables that could affect the dependent variables (slope) to identify the medium components. Independent factors and their levels were shown in Table 1. The design and the response of the observed dependent variables of PBD formulation were shown in Table 2 and Table 3. The relationships between response and variables were analyzed by ANOVA and the results were summarized in Table 4. The medium components were screened at the confidence level of 90% on the basis of their effects. EGF, FGF and linoleic acid were identified to have significant effects in response or cells growth. Therefore, we chose EGF, FGF and linoleic acid for further optimization. Confidence levels of the variables were higher than 90%, indicating that they had significant contributions than other medium components. The ANOVA showed the determination coefficient R^2^ of 96.71, implying that the model equation could explain 96.71% of the total variation.

**Table 4:** ANOVA for Plackett-Burman design.

Source	Sum of Squares	df	Mean Square	F Value	P-value Prob>F
**Model**	158.46	16	9.90	5.77	0.0871
**Transferrin**	0.20	1	0.20	0.12	0.7553
**Sodium selenite**	5.20	1	5.20	3.03	0.1800
**Sodium L-ascorbate**	3.70	1	3.70	2.16	0.2384
**Ferric citrate**	3.87	1	3.87	2.26	0.2301
**L-glutathione**	2.31	1	2.31	1.35	0.3297
**BSA**	0.032	1	0.032	0.019	0.9000
**EGF**	20.81	1	20.81	12.13	0.0400
**FGF**	38.64	1	38.64	22.52	0.0175
**Ethanolamine**	3.20	1	3.20	1.86	0.2654
**Linoleic acid**	35.91	1	35.91	20.93	0.0196
**Arachidonate**	0.072	1	0.072	0.042	0.8508
**Thioglycerol**	3.53	1	3.53	2.06	0.2471
**Hydrocortisone**	7.94	1	7.94	4.63	0.1206
**Yeast hydrolyzate**	1.46	1	1.46	0.85	0.4246
**Penicillin-Streptomycin**	10.37	1	10.37	6.04	0.0910
**Solution Succinic Acid**	21.22	1	21.22	12.36	0.0390
**Residual**	5.15	3	1.72		
**Cor Total**	163.61	19			

Determination coefficient R^2^ = 96.71; Adjusted determination R^2^ = 87.50; Coefficient of variation CV = 3.16%

### Optimization of selected medium components with CCD

In line with the results from Plackett-Burman design, EGF, FGF and linoleic acid were selected as most significant medium components and further optimized by use of response surface methodology with CCD. In this approach, the 20 runs were conducted in CCD experiments to determine the effect of independent variables on the response (cells density) along with the predicted values in Table 5. Using Design-Expert 7.0 to analyze the ANOVA of the model, which was performed to investigate the second-order response surface model, and the results were given in Table 5. The ANOVA demonstrated that the model was highly significant, as was evident from the P value (<0.0001) of the F-test. This high significance was also confirmed by the result of insignificant lack of fit (F = 1.25 and P>0.05, indicating that the ratio of the obtained equation to the actual fitting was small). The determination coefficient (R^2^) of 99.27 of the model suggested that model equation could explain 99.27% of the total variation, indicating a good agreement between predicated values and experimental data.

**Table 5:** ANOVA for central composite design of response surface methodology.

Source	Sum of Squares	df	Mean Square	F Value	P-value Prob>F
**Model**	1824.81	9	202.76	150.63	0.0001
**A-EGF**	87.15	1	87.15	64.75	0.0001
**B-FGF**	113.41	1	113.41	84.25	0.0001
**C-linoleic acid**	0.98	1	0.98	0.73	0.4134
**AB**	25.92	1	25.92	19.26	0.0014
**AC**	6.13	1	6.13	4.55	0.0587
**BC**	5.12	1	5.12	3.80	0.0797
**A^2^**	9.78	1	9.78	7.27	0.0225
**B^2^**	792.11	1	792.11	588.47	0.0001
**C^2^**	873.42	1	873.42	648.88	0.0001
**Residual**	13.46	10	13.46		
**Lack of Fit**	7.47	5	7.47	1.25	0.4070
**Pure Error**	5.99	5	5.99		
**Cor Total**	1838.27	19			

Determination coefficient R^2^ = 99.27; Adjusted determination Adj. R^2^ = 98.61; Coefficient of variation CV = 3.10%

The regression analysis was performed in the 20 groups, and the polynomial regression model was obtained by regression fitting. The regression equation coefficients were calculated and the data were fitted to a second-order polynomial equation:

$$Y=47.24-2.53A+2.88B-0.27C+1.80AB+0.88AC+0.80BC+0.82A_{2}-7.41B_{2}-7.79C_{2}$$

Where Y represented the response or cells density, and A, B and C were the coded values of EGF, FGF and linoleic acid, respectively.

**Fig 3:**
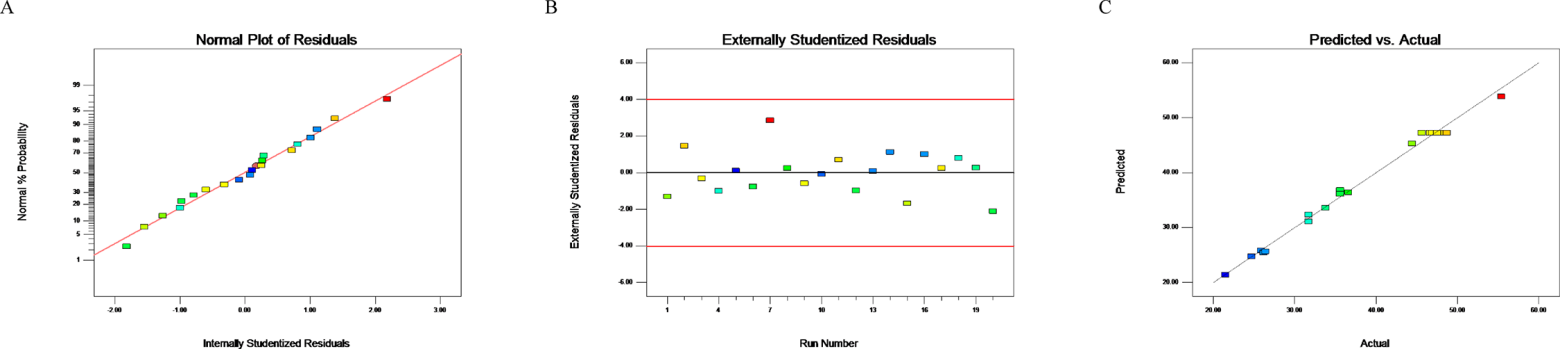
Residual diagnostic plots of the quadratic model. (A) The normal distribution of residuals. (B) Residuals analysis of the experiment. (C) Comparison between the experimental values and the predicted.

Diagnostic plots were used to analyze the model adequacy and clarify the sign of any problem in the experimental data. The plots of observed responses versus predicted responses were shown in Fig 3. The predicted values were in agreement with observed ones in the range of the operating variables, since they were located on both sides of s straight line. The normal probability plot of the studentized residuals was used to test the normality of residuals. A line pattern observed in this plot would clarify whether there was the sign of any problem in the experimental data. According to the plot of studentized residuals versus predicted values for evaluating the constant error, residual points were showed to be randomly scattered separately, indicating that the variance of the original observation was constant.

**Fig 4:**
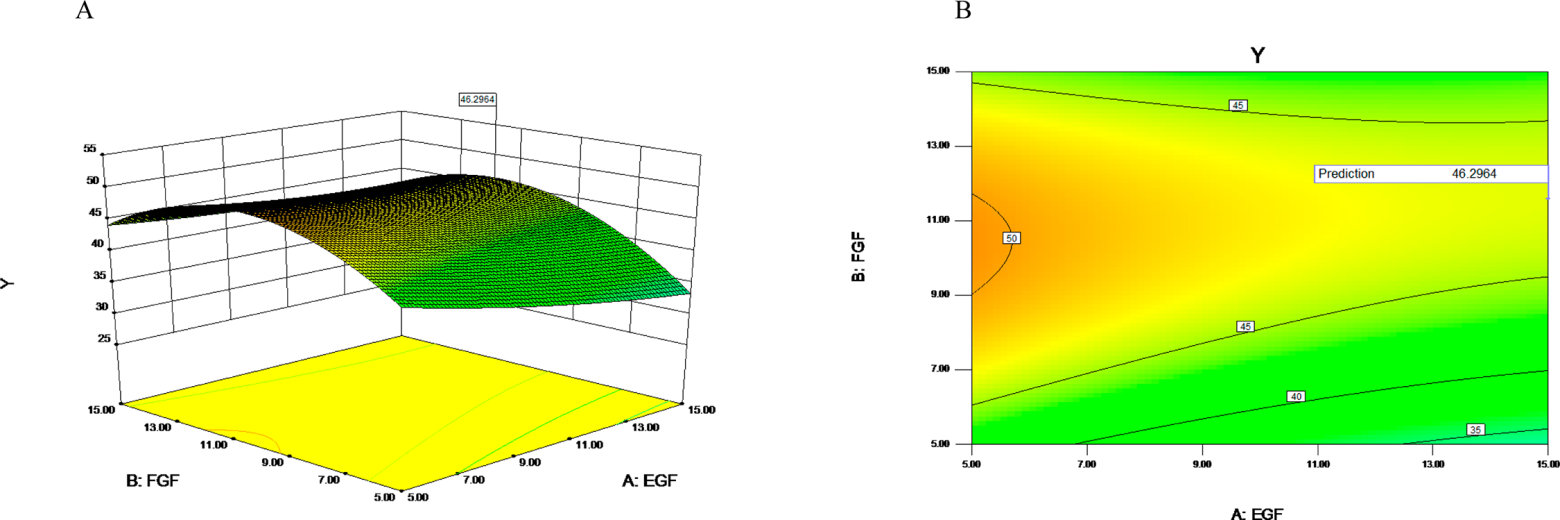
Response surface plot of the regression equation (EGF, FGF). The three-dimensional response surface plots (A) and contour charts (B) were used to illustrate the individual and interactive effects of EGF and FGF.

The three-dimensional (3D) response surface plots and contour charts were used to illustrate the individual and interactive effects of EGF, FGF and linoleic acid. Each 3D chart represented the effects of two variables while the rest one was maintained at the middle level. As shown in Fig 4, with the increasing concentration of FGF from 5ng/ml to 11.59ng/ml (coded value, -1 to 0.318) (X_B_ from -1 to 0.318), the response (Y, or cells density) significantly increased at the moderate concentration (10ng/ml) of EGF(coded value, 0); However, the response significantly decreased with the increasing concentration of FGF from 5ng/ml to 15ng/ml (X_B_ from 0.318 to 1) at the moderate concentration of EGF.

**Fig 5:**
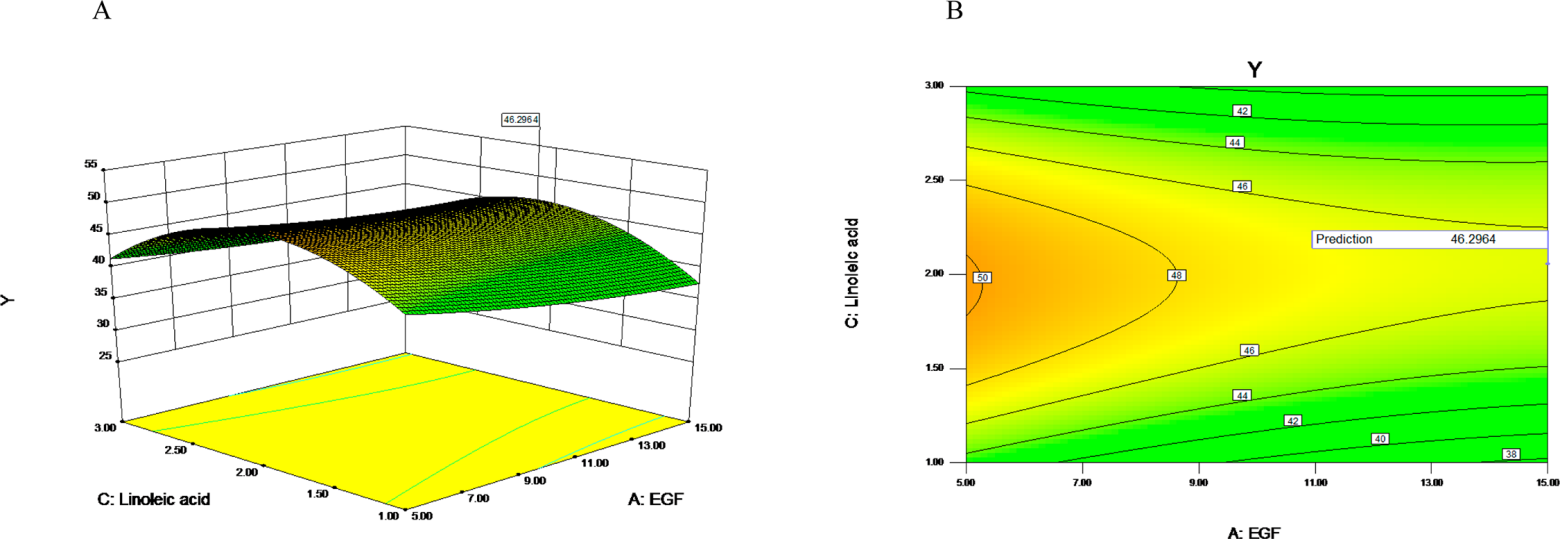
Response surface plot of the regression equation (EGF, linoleic acid). The three-dimensional response surface plots (A) and contour charts (B) were used to illustrate the individual and interactive effects of EGF and linoleic acid.

**Fig 6:**
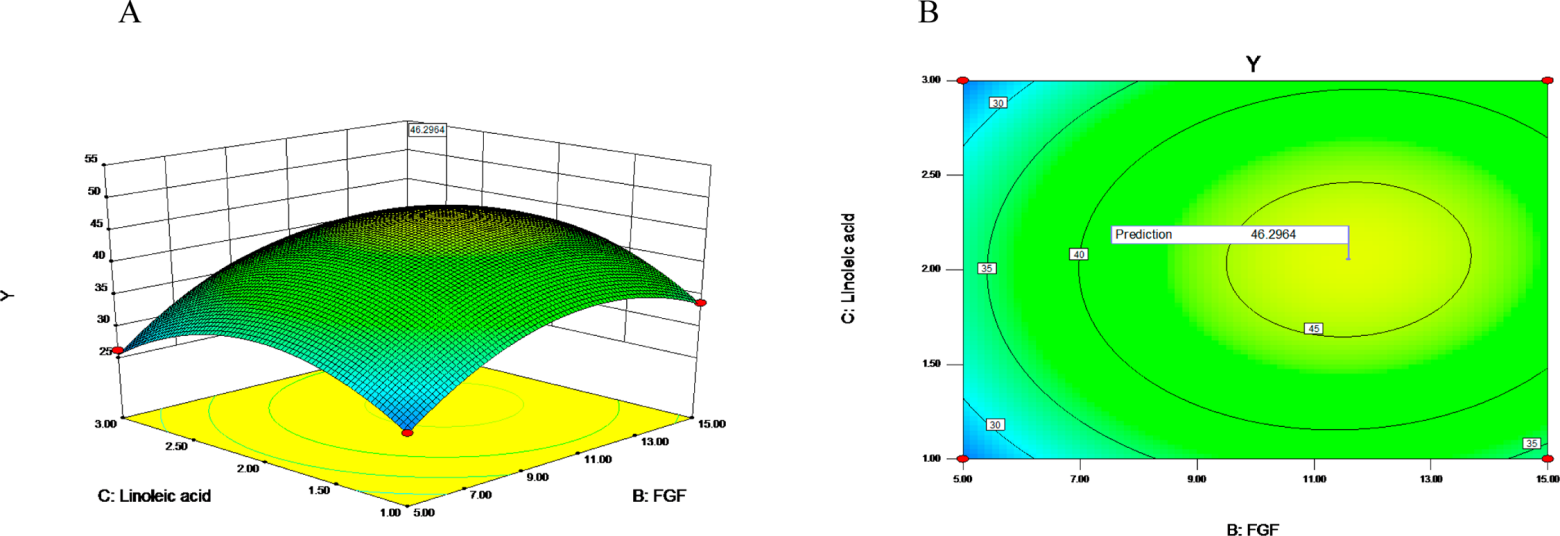
Response surface plot of the regression equation (linoleic acid, FGF). The three-dimensional response surface plots (A) and contour charts (B) were used to illustrate the individual and interactive effects of FGF and linoleic acid.

As shown in the Fig 5, with the increasing concentration of linoleic acid from 1mg/l to 2.06mg/l (coded value,-1 to 0.055) (X_C_ from -1 to 0.055) at the moderate concentration of EGF (coded value, 0) (X_A_ = 0), the response (Y) significantly increased at the beginning and then decreased. With the increasing concentration of EGF from 1.59ng/ml to 18.41ng/ml (coded value,-1 to 1) (X_A_ from -1 to 1), the response gradually decreased at the moderate concentration of linoleic acid (coded value, 0) (X_C_ = 0). Subsequently, the interaction between FGF and linoleic acid was investigated with EGF at the middle level. As shown in Fig 6, with the increasing concentration of linoleic acid from 1mg/l to 2mg/l (coded value, -1 to 0.055) (X_C_ from -1 to 0.055) at the moderate concentration of FGF (X_B_ = 0), the response significantly increased at the beginning and then decreased. The effect of the concentration of FGF on the response formed a parabola at the moderate concentration of linoleic acid, and the response value reached maximum when the FGF concentration was 11.59ng/ml (X_B_ = 0.318).

### Optimization and experimental validation

**Fig 7:**
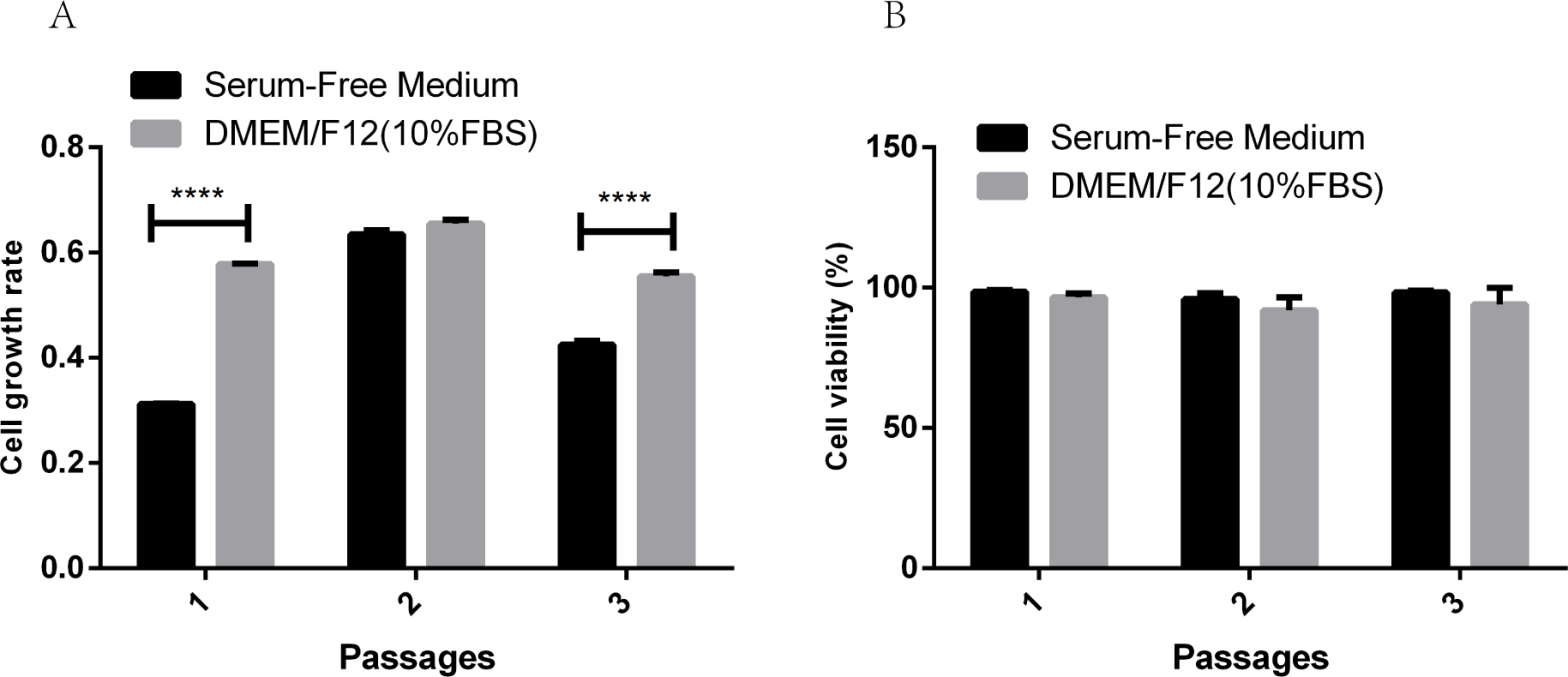
Verification of culture effect of serum-free medium. (A) The growth rates of PC-3 cells in the control groups and in the optimized groups. (B) The viability of PC-3 cells in the control groups and in the optimized groups.

According to the quadratic polynomial equation fitted with the experiment, when the response value reached to maximum (55.4), the concentrations of the three factors were optimized. The optimized concentrations were EGF (15ng/ml), FGF (11.59ng/ml) and linoleic acid (2.06mg/l), which were validated by long-term passage stability test of PC-3 cells. At the same time, PC-3 cells were cultured in growth medium (DMEM/F12) supplemented with 10% fetal bovine serum as the control in triplicate. As shown in Fig 7, the growth rates of PC-3 cells were 0.58, 0.65 and 0.55, respectively, in the control groups, while the growth rates of PC-3 cells were 0.31, 0.63 and 0.42, respectively, in the optimized groups. In addition, the viability of PC-3 cells in the control groups were 96.58%, 91.23% and 94.34%, while the viability of PC-3 cells in the optimized groups were 98.53%, 95.73% and 98.31%. Data were presented as mean ± SD. **** meant p<0.0001.

## Discussion

The biopharmaceutical industry is strongly motivated to develop high performing processes to meet increasing market demands and reduce manufacturing costs. Mammalian cell culture is one of the important areas in cell engineering. Many efforts have been made in the medium optimization. A well-balanced medium composition is essential for maximal viable cell density and productivity, two major elements of a fed-batch process. Usually, the medium optimization is performed sequentially due to the large number of experiments required for a simultaneous optimization. Here, we introduced the high-throughput RTCA in combination with statistical methodologies for the medium development of a fed-batch culture process of adherent cells. The xCELLigence RTCA is a non-invasive, small-scale, real-time monitoring and impedence-based biosensor system, which can measure cell viability, migration, growth, spreading and proliferation. Changes of cell morphology and behavior are continuously monitored in real time through the microelectronics located in the wells of RTCA E-plates [11,12].

By use of the technical advantages of RTCA, the proliferation characteristics of PC-3 cells were quantitatively analyzed to determine the best cell seeding density for screening serum-free media. Evaluation was to ensure that the cell proliferation curve had typical characteristics such as adaptation period, logarithmic growth phase and plateau stage, at the same time, taking into account that the cells from the serum culture into serum-free culture would lead to the selective pressure, which might cause some cell deaths and prolong the cell adaptation period. Finally, the cell seeding density of PC-3 cells was identified to be 2–4*10^5^ cell/L as shown in Fig 2. In addition, the four basic media DMEM, F12, DMEM/F12 and RPMI1640 were screened and then PC-3 cells were found to grow better in DMEM or DMEM/F12 with 5% FBS than in F12 or RPMI1640 with 5% FBS (Fig 1).

Glucose is an important source of energy and carbonaceous material for cell life activities. Normal cells are given priority to with aerobic metabolism of glucose, but not for the anaerobic fermentation. However, the cancer cells are just the opposite, given priority to with glucose-based anaerobic glycolysis, supplemented by aerobic glycolysis, and thus this phenomenon is called the “Warburg effect” [37, 38]. In the screening of PC-3 cell basal medium, PC-3 cells cultured in DMEM medium with high glucose were found to have a good proliferation performance. In line with the analysis of glucose metabolism in PC-3 cells, they were found to be metabolically dependent on glucose. Although the other three media had more comprehensive nutritional components, their glucose and amino acid concentrations were lower than the DMEM medium. In the serum-free culture nutritional deficiency in DMEM medium were noticed so that DMEM/F12 medium was chosen as the basal medium to optimize serum-free medium.

By use of Plackett-Burman design, a commonly used statistical screening method, we are able to do the minimum number of experiments to examine a large number of factors for the response of the significant value simultaneously. EGF, FGF, linoleic acid, penicillin-streptomycin solution and succinic acid were found to have significant effects on the response value, and the effects of EGF, FGF and linoleic acid were the most significant as shown in Tables 4 and 5 and from Fig 3 to Fig 7. EGFR is highly expressed on the surface of PC-3 cells, and EGF as growth factor can stimulate the rapid proliferation of PC-3 cells through its binding to EGFR [35, 39, 40]. Consistent with the optimization results from CCD, the rapid growth of PC-3 cells depended on high concentrations of EGF. FGF is a commonly used serum-free medium additive, which can stimulate DNA synthesis and cell proliferation in a variety of cells [26]. The unsaturated fatty acid linoleic acid as one of the commonly used in serum-free medium can promote cell proliferation and also promote cell expression of antibodies [24]. As demonstrated in this study, FGF and linoleic acid played a key role in cell proliferation of PC-3 cells.

In addition, there were significant differences of gene expression between the cells in the serum-free culture and those in the serum culture. The changes in gene expression in the cells were mainly related to cell metabolism, but the cell morphology and the original immunogenicity of its own didn’t change significantly [41]. Indeed, we didn’t notice any significant changes in terms of PC-3 immunogenicity, since we attempted to prepare large-scale culture of PC3 by use of our optimized media for the generation of hGM-CSF/hTNFα-surface-modified PC3 therapeutic vaccine for the prostate cancer [5].

## Conclusions

Our high-throughput optimization approach was a robust and rapid platform for the medium optimization of a fed-batch cell culture. Data analysis by simple ranking based on critical process outputs such as cell growth and viability, provided an easy and quick tool to identify the best medium formulations of good performance. On the other hand, statistical methodologies enabled a more in-depth analysis, allowing prediction of best medium formulations of good performance and identification of critical medium components for further optimization. Therefore, compared with traditional medium optimization strategies, the platform was efficient and cost-effective.
